# Supplementary material for: A direction-selective cortico-brainstem pathway adaptively modulates innate behaviors
Source: Nat Commun. 2023 Dec 20;14:8467. doi: 10.1038/s41467-023-42910-2 (PMC10733370; doi:10.1038/s41467-023-42910-2)
Supplement: Supplementary file 1 — Supplementary Information [file 41467_2023_42910_MOESM1_ESM.pdf]

Supplementary Information

A Direction-selective Cortico-Brainstem Pathway Adaptively Modulates Innate Behaviors

Jiashu Liu[1,2], Yingtian He[1,2], Andreanne Lavoie[1,2], Guy Bouvier[3], Bao-hua Liu*[1,2]
[1]Department of Biology, University of Toronto Mississauga, Mississauga, ON L5L 1C6, Canada
[2]Department of Cell and Systems Biology, University of Toronto, Toronto, ON M5S 3G5, Canada
[3]Université Paris-Saclay, CNRS, Institut des Neurosciences Paris-Saclay, 91400 Saclay, France
*Correspondence: baohua.liu@utoronto.ca

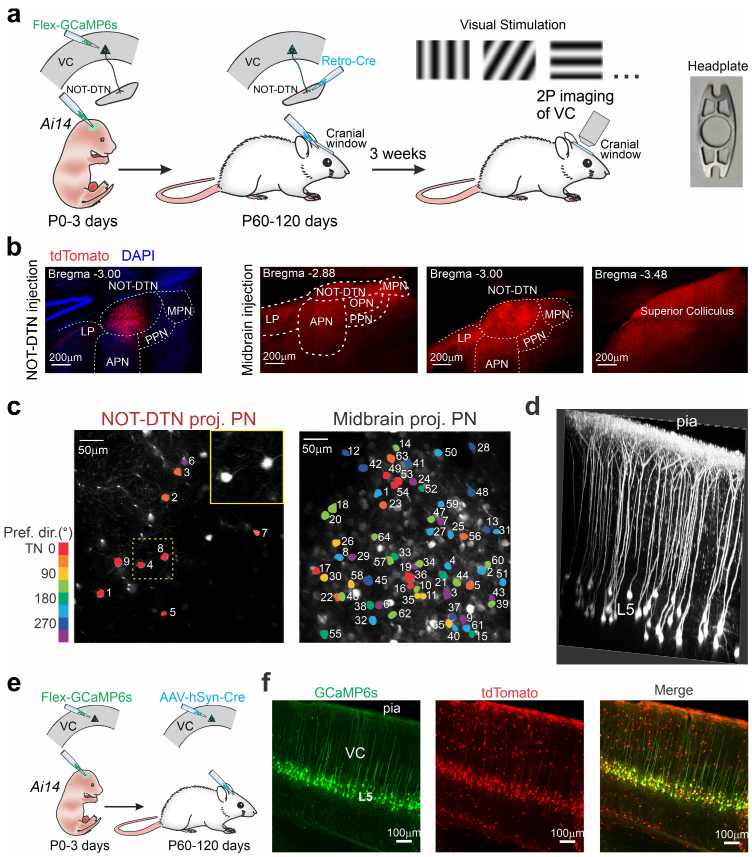

Supplementary Figure 1 | Two-photon imaging of L5 PNs in the visual cortex.

a, Left, schematic of experimental design. *Ai14*, tdTomato reporter mice; VC, visual cortex. Cre-dependent GCamP6s virus is injected in the visual cortex of *Ai14* pups. Retrograde Cre virus (Retro-Cre) is injected in the NOT-DTN of adult mice. Drifting sinusoidal gratings are presented to the contralateral eye during the recording. Right, the image of the headplate used for two-photon imaging. **b**, Coronal slices of the midbrain from mice with a local (left one) or diffusive (right three) injection of Retro-Cre virus to the NOT-DTN. Note that in the diffusive injection the virus spreads to the regions surrounding the NOT-DTN and thus labels midbrain-projecting pyramidal neurons (PNs). LP, lateral posterior thalamic nucleus; APN, anterior pretectal nucleus; PPN, posterior pretectal nucleus; MPN, medial pretectal nucleus; OPN, olivary pretectal nucleus. Boundaries are delineated according to Allen mouse brain atlas (atlas.brain-map.org). **c**, Example two-photon images of NOT-DTN-projecting (left) and midbrain-projecting (right) L5 PNs. Colors encode the preferred direction of neurons with DSI $\geqslant 0.1$. Inset, higher magnification of the enclosed area. TN, temporo-nasal. **d**, Side view of a 3D two-photon image of NOT-DTN-projecting PNs (520x520x673 μm, LxWxH). **e**, Schematic of viral injection for imaging general L5 PNs. Note that Cre virus is injected at the border of L5 and L6. **f**, Coronal slice of visual cortex. Note that GCaMP6s is strongly expressed mainly in L5 PNs. Schematics in **a** and **e** are adapted from Liu *et al.* (2016)[2].

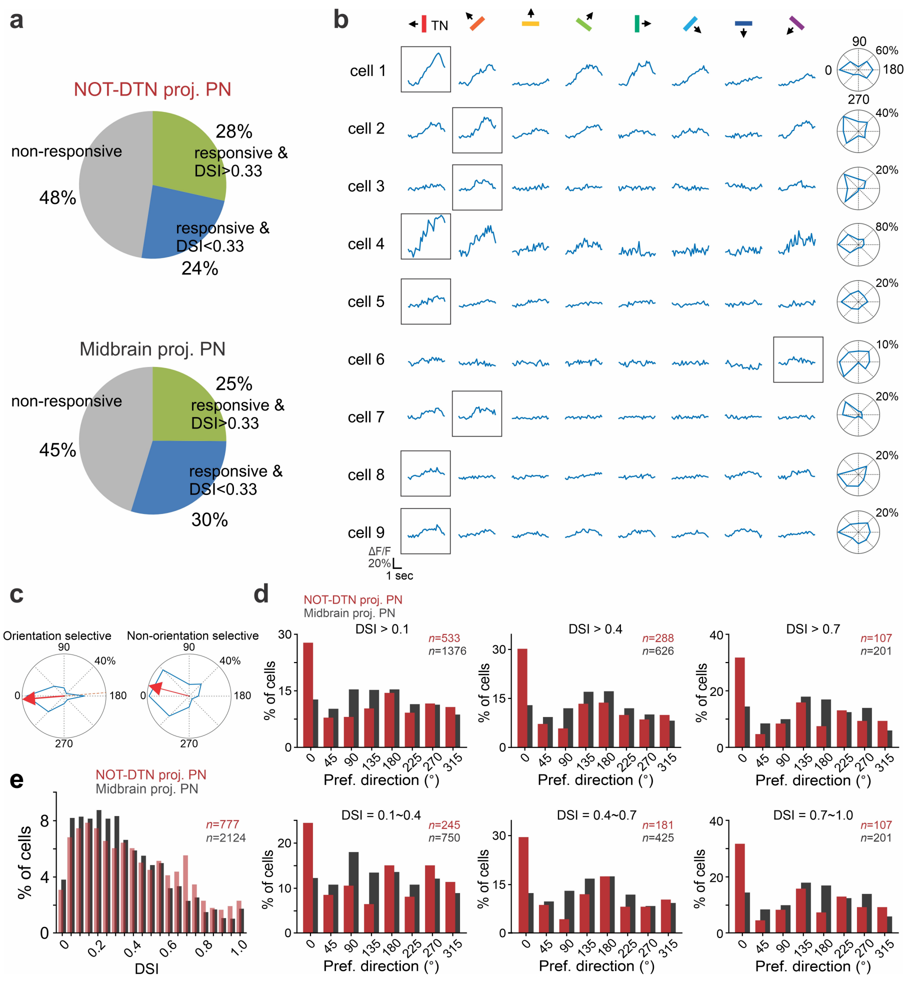

Supplementary Figure 2 | Direction selectivity of two corticofugal populations in the visual cortex.

a, Pie charts of visually responsive and direction-selective neurons in NOT-DTN-projecting or midbrain-projecting populations. PN, pyramidal neuron. **b**, Example NOT-DTN-projecting PNs in **Supplementary Figure 1c left**. Left, trial-averaged calcium responses (ΔF/F) sorted according to the directions of moving gratings (indicated by arrows and colored bars). Open boxes, maximum responses. Right, polar plots of direction tuning curves. Cell 4 is the same neuron in **Figure 1c**. TN, temporo-nasal. **c**, Preferred directions of an orientation selective neuron (left) or non-orientation selective neuron (right). See details in methods. **d**, Histograms of the preferred direction for PNs with different ranges of DSI. Note that NOT-DTN-projecting population (red) has much more neurons with the bias towards temporo-nasal direction (0°) than midbrain-projecting population (black) (*P*<1e-06 for DSI>0.1, 1e-06 for DSI>0.4, 3e-04 for DSI>0.7, 1e-05 for DSI=0.1~0.4, 1e-06 for DSI=0.4~0.7, and 3e-04 for DSI=0.7~1; one-sided randomization test). **e**, Histograms of DSI. Note that higher percentage of NOT-DTN-projecting PNs have DSI ⩾ 0.33 than midbrain-projecting PNs (54% vs 45%).

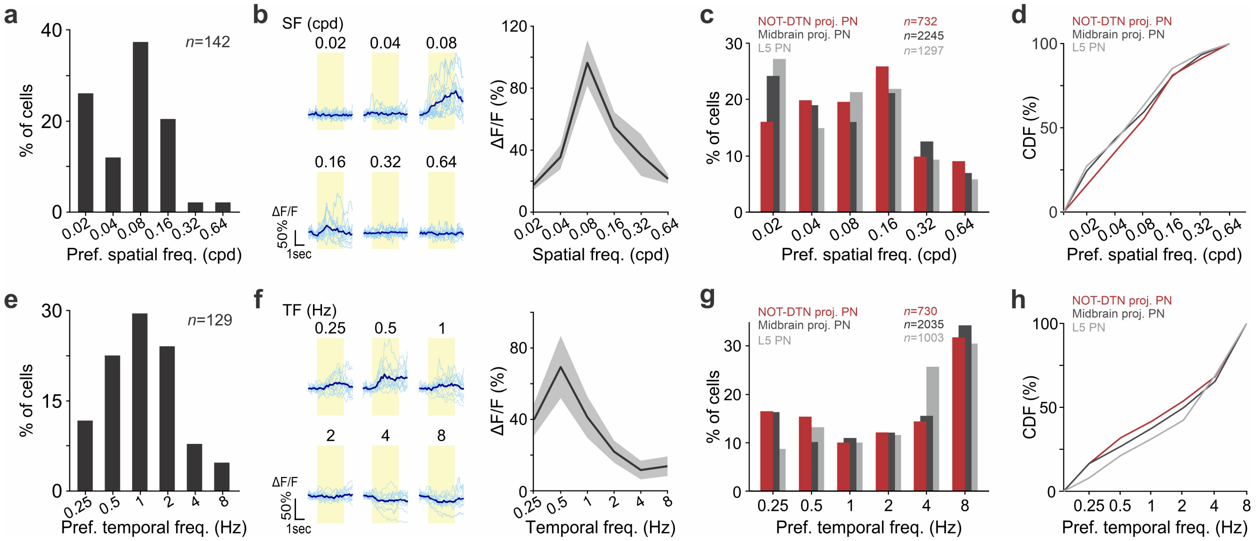

Supplementary Figure 3 | Comparison in spatial frequency tuning and temporal frequency tuning between three L5 PN populations.

a, Histograms of preferred spatial frequency of NOT-DTN neurons. **b**, Spatial frequency tuning of an example NOT-DTN-projecting pyramidal neuron (PN). Left, calcium responses (ΔF/F) sorted according to various spatial frequencies. Light blue traces, individual trials; dark blue traces, average. Yellow shades indicate the duration of visual stimulation. Right, spatial frequency tuning curve of the example on the left. Thickness, s.e.m. SF, spatial frequency. **c**–**d**, Histogram (**c**) and cumulative distribution function (CDF, **d**) of preferred spatial frequency among three L5 PN populations. Note that more neurons prefer intermediate SF in NOT-DTN-projecting population than midbrain-projecting population (*P*=4e-04, two-sided Kolmogorov-Smirnov (KS) test) or L5 PNs (*P*= 6.5e-06, two-sided KS test). **e**–**h**, Temporal frequency (TF) tuning of NOT-DTN neurons (**e**) and three L5 PN populations (**f**–**h**). Data are presented as in **a**–**d**. Note that more neurons prefer low TF in NOT-DTN-projecting population than midbrain-projecting population (*P*=0.05, two-sided KS test) or L5 PNs (*P*= 1.3e-05, two-sided KS test).

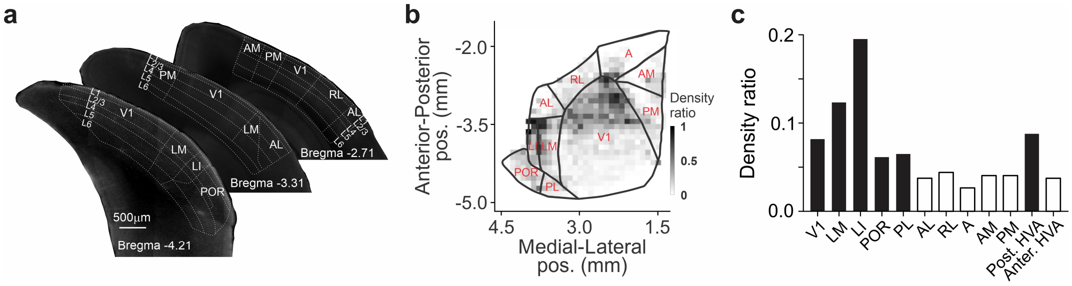

Supplementary Figure 4 | Density ratio of NOT-DTN-projecting PNs to midbrain-projecting PNs in visual areas.

a, Coronal slices containing the visual cortex contralateral to the hemisphere where Retro-Cre virus was injected into the NOT-DTN. Note that no neuron is labeled in the contralateral visual areas. **b**, Map of density ratio in visual areas. Boundaries between individual areas are determined according to the Allen mouse brain atlas (atlas.brain-map.org). **c**, Average density ratio of individual visual areas. Solid bars, V1 and posterior higher visual areas (HVAs); open bars, anterior HVAs. PM, posteromedial; AM, anteromedial; A, anterior; RL, rostrolateral; AL, anterolateral; LI, laterointermediate; LM, lateromedial; POR, postrhinal; PL, posterolateral.

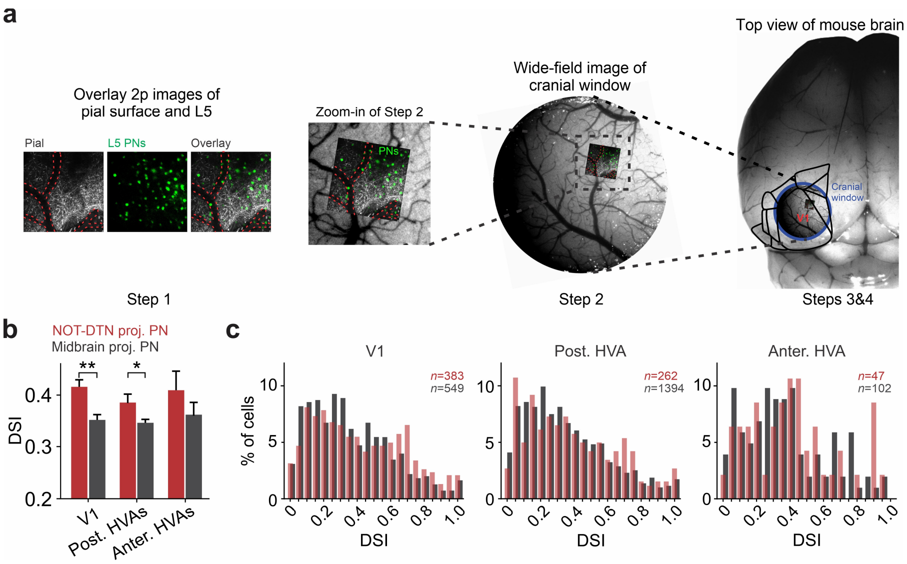

Supplementary Figure 5 | Direction selectivity of corticofugal PNs located in different visual areas.

a, Identification of visual areas where individual neurons in two-photon imaging reside. Step 1: Overlay the two-photon images of the pial surface and L5 pyramidal neurons (PNs) taken from the same cortical location. Blood vessels in the image of the pial surface are highlighted by red dashed lines. Step 2: Superimpose the two-photon image in step 1 on top of a widefield image of the cranial window according to the blood vessel pattern. Step 3: Superimpose the widefield image of the cranial window on a top-view image of the whole mount brain according to the blood vessel pattern. Step 4: Overlay the outlines of visual areas (Allen Mouse Brain Atlas, atlas.brain-map.org) over the top view of the mouse brain based on the reference coordinates. **b**–**c**, Summary (**b**) and histograms (**c**) of DSI of the two corticofugal populations from different visual areas. *, *P*=0.02; **, *P*=1.9e-4; one-sided Wilcoxon rank-sum test. Data shown as mean ± s.e.m. Note that higher percentage of NOT-DTN-projecting PNs have DSI $\geqslant$ 0.33 than midbrain-projecting PNs in all groups (55% vs 46% for V1, 52% vs 45% for posterior higher visual areas (HVAs), 62% vs 50% for anterior HVAs). The sample sizes in **c** are the same as the ones of the corresponding groups in **b**.

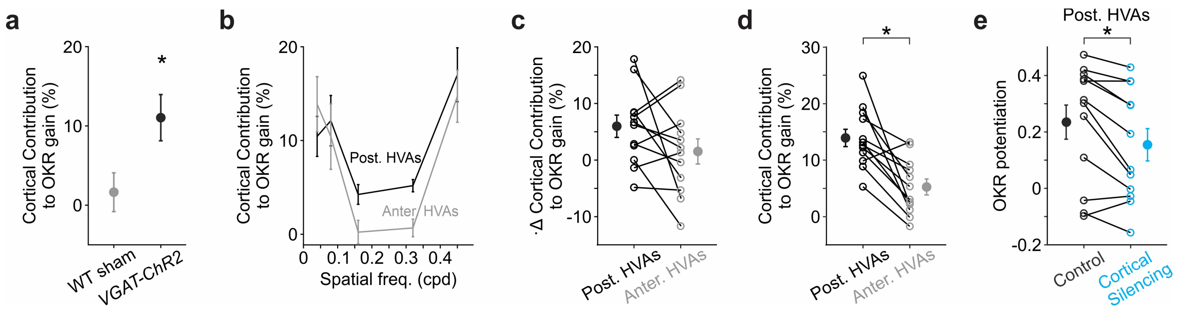

Supplementary Figure 6 | Cortical contribution of HVAs to OKR behavior.

a, Comparison in the effect of illuminating the visual cortex on OKR gain between wild-type sham mice (n=15) and *VGAT-ChR2* mice (n=16). Note that OKR gain is reduced in *VGAT-ChR2* mice (*, P=0.002, one-sided Wilcoxon signed-rank test), but not in wild-type sham mice. **b**, Averaged spatial frequency tuning curves of the contribution of posterior or anterior higher visual areas (HVAs) to OKR gain (n=11 mice). **c**, Summary of the change in cortical contribution to OKR gain induced by continuous OKR stimulation. Note that the rise in cortical contribution from posterior HVAs exceeds that originating from anterior HVAs (n=12 mice, P=0.1, one-sided Wilcoxon signed-rank test) after inducing OKR potentiation. **d**, Summary of the contribution of posterior or anterior HVAs to OKR gain after the induction of OKR potentiation (n=12 mice; *, P=4.9e-4, one-sided Wilcoxon signed-rank test). **e**, Summary of OKR potentiation with (cortical silencing) or without (control) silencing posterior HVAs (n=12 mice, P=0.005, one-sided Wilcoxon signed-rank test). Data in **a–e** shown as mean ± s.e.m.

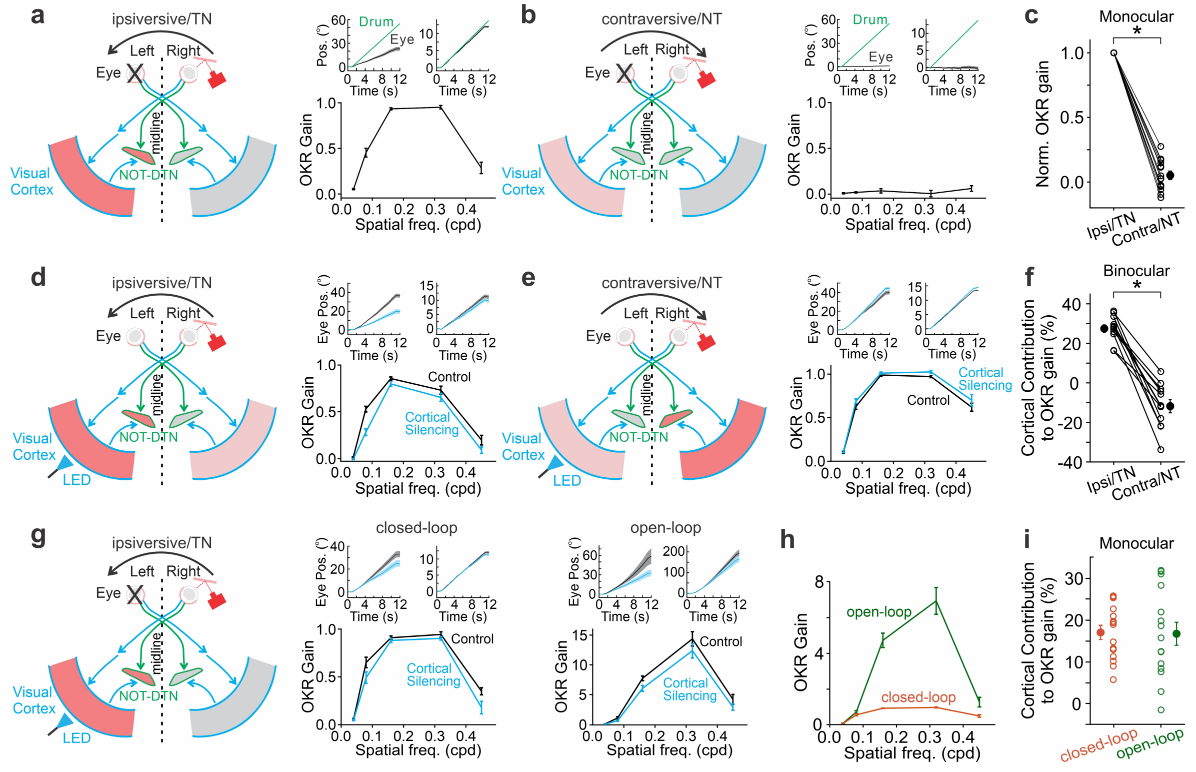

Supplementary Figure 7 | Direction-dependent cortical contribution to OKR behavior.

a, Monocular OKR evoked by temporo-nasal motion with respect to the open eye (equivalently ipsiversive to the contralateral NOT-DTN and visual cortex). Left, schematic of the circuits. Dark pink represents the strong activation of the NOT-DTN and NOT-DTN-projecting pyramidal neurons (PNs) by temporo-nasal (TN) visual motion. Note that the uncrossed retinal ganglion axons to dLGN are omitted for simplicity. Right, spatial frequency tuning curves of OKR gain from one mouse (*n*=24 trials). Top traces, averaged OKR trajectories (black, *n*=24 trials) at spatial frequencies 0.08 cpd (left) and 0.32 cpd (right) vs drum grating trajectory (green). Note that the nystagmus has been removed to show the slow eye movement. Thickness, s.e.m. **b**, Monocular OKR evoked by naso-temporal motion with respect to the open eye (equivalently contraversive to the contralateral NOT-DTN and visual cortex). Data are present as in **a**. Light pink represents the weak activation of NOT-DTN-projecting PNs by naso-temporal visual motion. Note that naso-temporal visual motion does not activate the NOT-DTN. **c**, Summary of normalized gain of monocular OKR evoked by temporo-nasal (Ipsi/TN) or naso-temporal (Contra/NT) directions. *n*=15 mice. *, *P*=3e-5, one-sided Wilcoxon signed-rank test. **d**–**f**, Unilateral cortical contribution to binocular OKR behavior evoked by drum gratings moving ipsiversively (**d**) or contraversively (**e**) with respect to the side of cortical silencing (equivalently temporo-nasal or naso-temporal to the contralateral eye). Data are present as in **a**–**c**. *n*=12 mice. *, *P*=2.4e-4, one-sided Wilcoxon signed-rank test. Blue and black curves, with and without cortical silencing, respectively. Ipsi/TN, P=2.4e-4; Contra/NT, P=0.002; one-sided Wilcoxon signed-rank test. **g**, Unilateral cortical contribution to monocular OKR behavior elicited by temporo-nasal (ipsiversive) motion in closed-loop or open-loop conditions. Left, schematic of the circuits. Right, spatial frequency tuning curves from example one mouse. Data are present as in **a**. Blue and black curves, with and without cortical silencing, respectively. **h**, Population spatial frequency tuning curves of OKR gain in closed-loop and open-loop conditions (*n*=13 mice). Note that the OKR gain in open-loop condition is much higher than 1. **i**, Summary of cortical contribution to OKR gain in two conditions (*n*=13

mice). Closed-loop, P=3e-5; Open-loop, P=6e-5; one-sided Wilcoxon signed-rank test. Data in **a**–**i** shown as mean ± s.e.m.

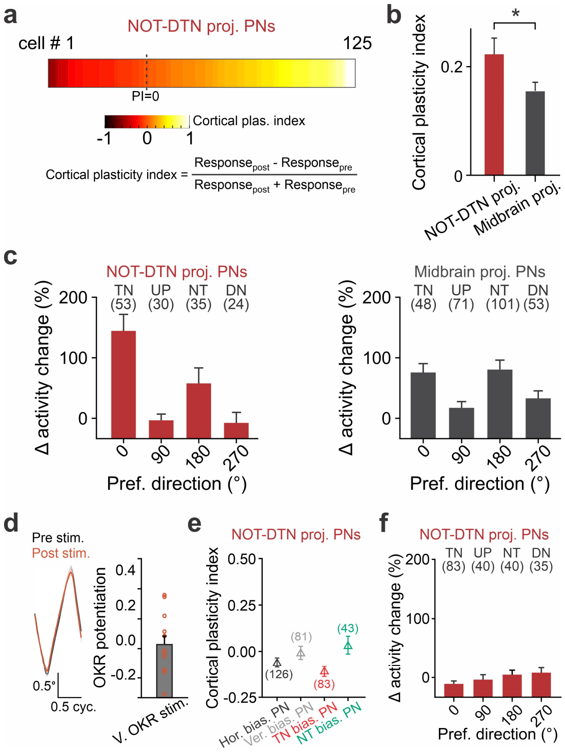

Supplementary Figure 8 | Quantification of cortical plasticity associated with OKR potentiation.

a, Heatmap of cortical plasticity index of NOT-DTN-projecting pyramidal neurons (PNs, *n*=125 cells). Calcium responses evoked by preferred directions are used. Dashed line, no change in calcium responses following OKR potentiation (equivalent to cortical plasticity index=0). **b**, Summary of cortical plasticity index of responses evoked by preferred directions. *, *P*=0.03, one-sided Wilcoxon rank-sum test ; *n*=125 vs 281 neurons, NOT-DTN-projecting vs midbrain-projecting PNs. **c**, Comparison of percentage change in calcium responses following OKR potentiation among four direction-biased PN populations (TN/UP/NT/DN biased PNs). Responses evoked by temporo-nasal direction are used. Note that the temporo-nasally biased neurons in NOT-DTN-projecting population (but not in midbrain-projecting population) show much stronger cortical plasticity than neurons preferring other directions (direction-selective plasticity). TN, temporo-nasal; NT, naso-temporal; UP, up; DN, down. The numbers in brackets, sample sizes. **d**, Left, cycle-averaged horizontal OKR trajectories from one example animal before (Pre-stim.) and after (Post-stim.) continuous vertical OKR stimulation. Thickness, s.e.m. Right, Summary of horizontal OKR potentiation after the exposure to continuous vertical OKR stimulation (V. OKR stim., *n*=13 mice). **e**, Summary of plasticity index of temporo-nasally evoked activity among NOT-DTN-projecting PNs that prefer the horizontal motions (Hor. bias. PN, 0° and 180°), vertical motions (Ver. bias. PN, 90° and 270°), temporo-nasal motion (TN bias. PN), or naso-temporal motion (NT bias. PN). The numbers in brackets, sample sizes. **f**, Percentage change in calcium responses following continuous vertical OKR stimulation among four direction-biased PN populations. Responses evoked by temporo-nasal direction are used. The numbers in brackets, sample sizes. Data in **b**–**f** shown as mean ± s.e.m.

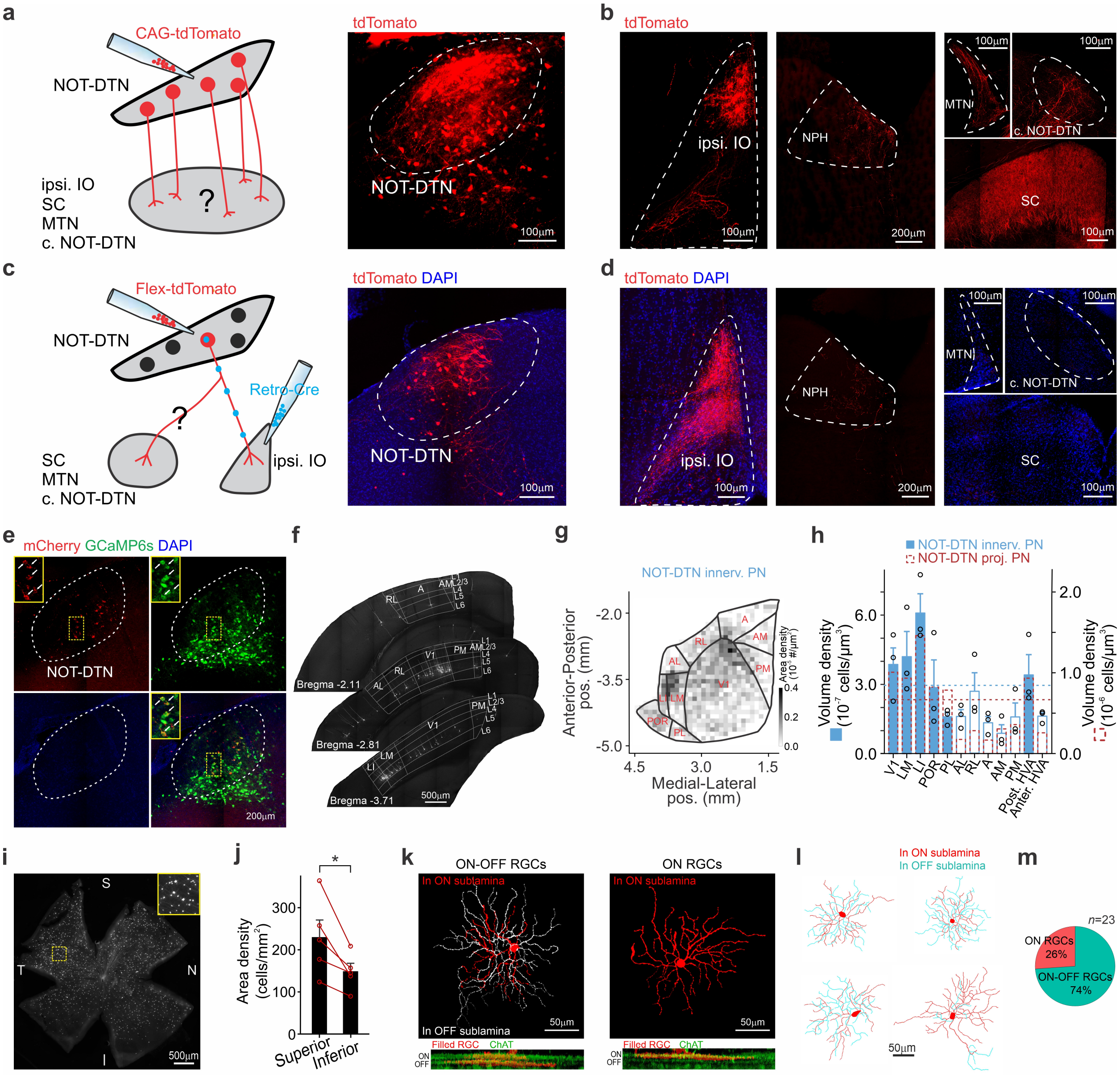

Supplementary Figure 9 | NOT-DTN neurons that project to IO form a distinct population.

a, Left, schematic of identifying the projection targets of NOT-DTN neurons. Right, coronal slice of the NOT-DTN. **b**, Coronal slices of brainstem structures downstream of NOT-DTN (*n*=1). Ipsi. IO, ipsilateral inferior olive; NPH, nucleus prepositus hypoglossi; MTN, medial terminal nucleus; c. NOT-DTN, contralateral NOT-DTN; SC, superior colliculus. **c**, Left, schematic of retrogradely labeling NOT-DTN neurons that project to the IO. Right, coronal slice of the NOT-DTN. **d**, Coronal slices of brainstem structures downstream of NOT-DTN (*n*=4). Note that red axons exist densely in ipsilateral IO, sparsely in NPH, but not in other downstream structures. **e**, Coronal slice of the NOT-DTN from rabies trans-synaptic tracing experiments. Inset, higher magnification of the enclosed area. Arrows point to the starter cells (IO-projecting NOT-DTN neurons), which express both mCherry and GCaMP6s. Note that the neurons expressing only GCaMP6s are trans-synaptically labeled presynaptic neurons. **f**, Spatial distribution of cortical PNs that synapse on IO-projecting NOT-DTN neurons (NOT-DTN-innervating PNs) on three coronal

slices containing visual cortex. Boundaries between primary and higher visual areas are delineated according to Allen mouse brain atlas (atlas.brain-map.org). **g**, Area density map of NOT-DTN-innervating PNs (*n*=3 mice). **h**, Volumetric density of NOT-DTN-innervating PNs (blue) in individual visual areas. Dashed lines indicate average densities across the visual cortex. Solid Bars, V1 and posterior HVAs; open bars, anterior HVAs. PM, posteromedial; AM, anteromedial; A, anterior; RL, rostrolateral; AL, anterolateral; LI, laterointermediate; LM, lateromedial; POR, postrhinal; PL, posterolateral. Open circles, data from individual mice in which NOT-DTN-innervating PNs were trans-synaptically labeled. For comparison purpose, data of the NOT-DTN-projecting PNs from **Fig. 2c top** is superimposed (red dashed bars). **i**, Example retina image from rabies trans-synaptic tracing experiments, in which the retinal ganglion cells (RGCs) synapsing on IO-projecting NOT-DTN neurons are labeled by GCaMP6s. Inset, higher magnification of the enclosed area. Note that the density of GCaMP6s positive RGCs differs between superior and inferior sectors. S, superior; I, inferior; T, temporal; N, nasal. **j**, Area density of NOT-DTN-innervating RGCs in superior or inferior portions of the retina (*n*=5 mice). *, *P*=0.03, one-sided Wilcoxon signed-rank test. **k**, Example NOT-DTN-innervating RGCs. Dendrites stratifying in the ON or OFF sublaminae of the inner plexiform layer are shown in red or white, respectively. Top, en face view; bottom, side view. **l**, Morphological reconstructions of more example NOT-DTN-innervating RGCs. Note that the bottom right is an ON RGC, while the rest are ON-OFF RGCs. **m**, Pie chart of the percentage of ON RGCs and ON-OFF RGCs that synapse on IO-projecting NOT-DTN neurons. Data in **h** and **j** shown as mean ± s.e.m. Schematics in **a** and **c** are adapted from Liu *et al.* (2016)[2].

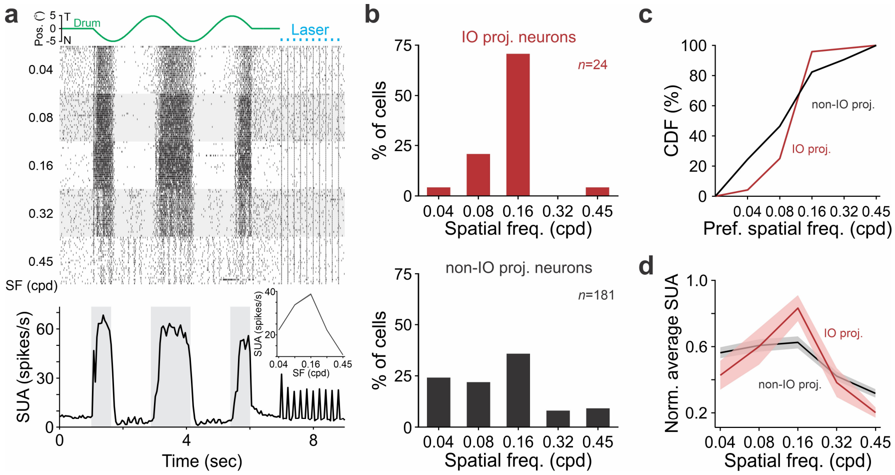

Supplementary Figure 10 | IO-projecting NOT-DTN neurons prefer intermediate spatial frequencies.

a, Spatial frequency (SF) tuning of an example IO-projecting NOT-DTN neuron. Top, trajectory of moving grating (green trace) and timing of laser pulses (blue dots, duration=5ms, frequency=5Hz). Middle, raster plots of single-unit activity (SUA) sorted by the spatial frequency of moving gratings. Bottom, post-stimulus time histogram (PSTH) of the same neuron. Shaded areas indicate the temporo-nasal phase of drum trajectory. Inset, spatial frequency tuning curve. Thickness, s.e.m. **b**, Histograms of preferred spatial frequency of IO-projecting (top) or non-IO-projecting NOT-DTN neurons (bottom). Note that higher percentage of IO-projecting NOT-DTN neurons prefer intermediate SF than non-IO-projecting ones (P=0.0012, one-sided randomization test). **c**, Cumulative distribution function (CDF) of preferred spatial frequency of the two NOT-DTN populations (n=24 vs 181 neurons, IO-projecting vs non-IO-projecting). **d**, Population-averaged normalized curves of spatial frequency tuning (same number of neurons as in **c**). The tuning curves of individual units are normalized against the maximum absolute values of their evoked firing rates. Thickness, s.e.m.

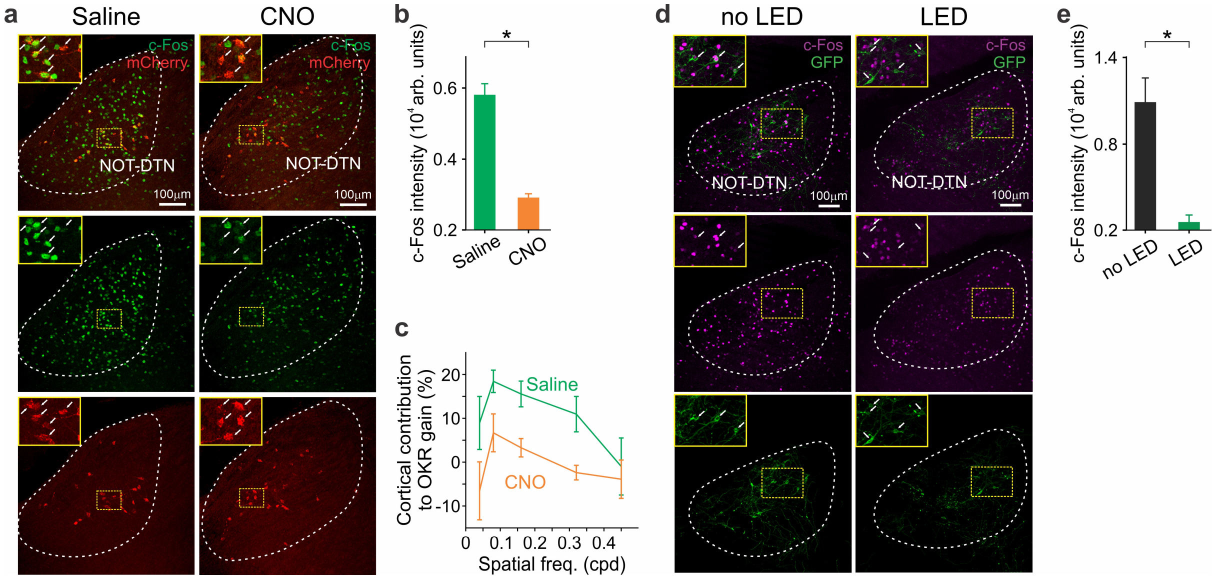

Supplementary Figure 11 | Efficiency of chemogenetically or optogenetically silencing IO-projecting NOT-DTN neurons.

a–**c**, Chemogenetic silencing of IO-projecting NOT-DTN neurons. **a**, c-Fos immunostaining of coronal slices containing NOT-DTN. Before OKR stimulation, either saline (left) or CNO (right) are administered by intraperitoneal injection. Insets, higher magnification of the enclosed areas. Arrows point to mCherry positive IO-projecting NOT-DTN neurons. Note that those neurons have higher c-Fos expression with saline injection than with CNO injection. **b**, Summary of c-Fos fluorescence intensity of mCherry positive IO-projecting NOT-DTN neurons (n=111 vs 296, saline vs CNO, P=2.9e-22, one-sided Wilcoxon rank-sum test). **c**, Population spatial frequency tuning curves of cortical contribution to OKR gain with intact (saline) or silenced (CNO) IO-projecting NOT-DTN neurons (n=12 mice). **d–e**, Optogenetic silencing of IO-projecting NOT-DTN neurons. **d**, c-Fos immunostaining of coronal slices containing NOT-DTN. Insets, higher magnification of the enclosed areas. Arrows point to GFP positive IO-projecting NOT-DTN neurons. Note that those neurons have higher c-Fos expression without LED illumination than with LED illumination. **e**, Summary of c-Fos fluorescence intensity of GFP positive IO-projecting NOT-DTN neurons (n=125 vs 78 neurons, with vs without LED, P=1.4e-7, one-sided Wilcoxon rank-sum test). Data in **b**,**c**,**e** shown as mean ± s.e.m.

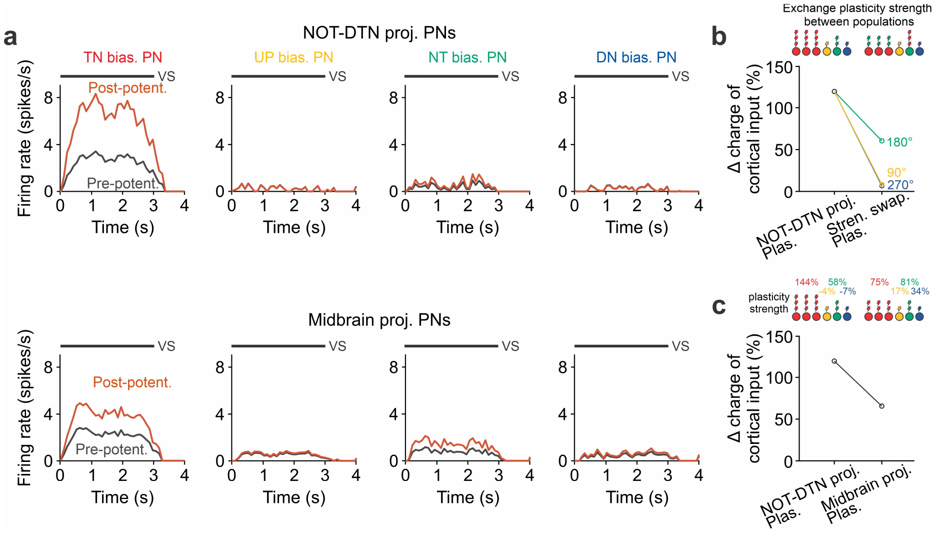

Supplementary Figure 12 | Modeling the integration of cortical input in the NOT-DTN.

a, Average firing rates of spiking activity evoked by the temporo-nasal visual motion among four direction-biased corticofugal populations (TN/UP/NT/DN biased PNs). TN, temporo-nasal; NT, naso-temporal; UP, up; DN, down; PN, pyramidal neuron; VS, visual stimulation. They were estimated from calcium responses of PNs in **Figures 1d&e**. Pre-potent., before OKR potentiation; post-potent., after OKR potentiation. Black bar, the duration of visual stimulation. **b**, Percentage change in cortical input (charge) provided by NOT-DTN-projecting population when the profile of its cortical plasticity strength is used (left) or when the plasticity strength value of temporo-nasally biased PNs is swapped with those of non-temporo-nasally biased neurons (right). **c**, Percentage change in cortical input (charge) provided by NOT-DTN-projecting population when its plasticity profile (left) or the plasticity profile of midbrain-projecting population (right) is used.
