# Supplementary material for: A direction-selective cortico-brainstem pathway adaptively modulates innate behaviors
Source: Nat Commun. 2023 Dec 20;14:8467. doi: 10.1038/s41467-023-42910-2 (PMC10733370; doi:10.1038/s41467-023-42910-2)
Supplement: Supplementary file 3 — Reporting Summary [file 41467_2023_42910_MOESM3_ESM.pdf]

nature portfolio

Corresponding author(s): Baohua Liu

Last updated by author(s): Oct 10, 2023

Reporting Summary

Nature Portfolio wishes to improve the reproducibility of the work that we publish. This form provides structure for consistency and transparency in reporting. For further information on Nature Portfolio policies, see our Editorial Policies and the Editorial Policy Checklist.

Statistics

For all statistical analyses, confirm that the following items are present in the figure legend, table legend, main text, or Methods section.

n/a	Confirmed	
☐	☒	The exact sample size (n) for each experimental group/condition, given as a discrete number and unit of measurement
☐	☒	A statement on whether measurements were taken from distinct samples or whether the same sample was measured repeatedly
☐	☒	The statistical test(s) used AND whether they are one- or two-sided<br>*Only common tests should be described solely by name; describe more complex techniques in the Methods section.*
☒	☐	A description of all covariates tested
☐	☒	A description of any assumptions or corrections, such as tests of normality and adjustment for multiple comparisons
☐	☒	A full description of the statistical parameters including central tendency (e.g. means) or other basic estimates (e.g. regression coefficient) AND variation (e.g. standard deviation) or associated estimates of uncertainty (e.g. confidence intervals)
☐	☒	For null hypothesis testing, the test statistic (e.g. F, t, r) with confidence intervals, effect sizes, degrees of freedom and P value noted<br>*Give P values as exact values whenever suitable.*
☒	☐	For Bayesian analysis, information on the choice of priors and Markov chain Monte Carlo settings
☒	☐	For hierarchical and complex designs, identification of the appropriate level for tests and full reporting of outcomes
☒	☐	Estimates of effect sizes (e.g. Cohen's d, Pearson's r), indicating how they were calculated

Our web collection on statistics for biologists contains articles on many of the points above.

Software and code

Policy information about availability of computer code

Data collection	ScanImage 2018 and Matlab R2018b were used for two-photon imaging. Customized codes running in LabVIEW 2014 were used to record eye movements in the optokinetic reflex behavior. Intan RHD 2000 interface V1_5_2 was used to record extracellular spiking activity. Customized codes running in Psychopy 1.90.2 (python 2 based) were used to generate visual stimulation. Zeiss Zen 2.3 SP1 was used for confocal imaging. AxioVision 4.8.2 was used for wide-field imaging. Olympus VS200 Slide Scanner (OlyVIA 3.4.1) was used to document the NOT-DTN innervating PNs labeled by rabies virus-based transsynaptic tracing.
Data analysis	Suite2p v0.7.1 (open source) was used to analyze data from two-photon calcium imaging. Kilosort2 and phy 2.0 beta 1 (open source) were used to sort and cluster spike waveforms in extracellular recording. Customized codes running in MATLAB R2018a were used to quantify calcium responses, OKR amplitudes, and visual feature selectivity, to model cortical innervation to the brainstem, to make figures and to perform statistic tests. ImageJ was used to process and analyze confocal images. R (version 3.6.1) was used to perform linear regression with the maximum likelihood estimation.<br><br>Custom codes used in data analysis and modeling are available on a publicly available repository GitHub at https://github.com/liulabutm/Codes-of-corticofugal-paper.git.

For manuscripts utilizing custom algorithms or software that are central to the research but not yet described in published literature, software must be made available to editors and reviewers. We strongly encourage code deposition in a community repository (e.g. GitHub). See the Nature Portfolio guidelines for submitting code & software for further information.

Data

Policy information about availability of data

All manuscripts must include a data availability statement. This statement should provide the following information, where applicable:

- Accession codes, unique identifiers, or web links for publicly available datasets
- A description of any restrictions on data availability
- For clinical datasets or third party data, please ensure that the statement adheres to our policy

All data analyzed for this study are presented in the article, supplementary figures, and source data. Source data are provided with this paper. The Allen Mouse Brain Atlas (https://atlas.brain-map.org/) was used to determine the boundaries of visual areas. Paxinos and Franklin's The Mouse Brain in Stereotaxic Coordinates (Elsevier, 2012) was used to determine the boundaries of subcortical structures. Because the raw and pre-processed datasets that support the findings of this study within the article and its supplementary materials are huge and presented in highly diverse formats, they are available from the corresponding author upon request.

Research involving human participants, their data, or biological material

Policy information about studies with human participants or human data. See also policy information about sex, gender (identity/presentation), and sexual orientation and race, ethnicity and racism.

Reporting on sex and gender	Since there was no report of sex dimorphism in the cortical modulation of OKR, the sex was not considered in the experimental design. Mice of both sexes were used for experiments. The sex of mice used in each experiment was included in the Source Data file.
Reporting on race, ethnicity, or other socially relevant groupings	n/a
Population characteristics	n/a
Recruitment	n/a
Ethics oversight	n/a

Note that full information on the approval of the study protocol must also be provided in the manuscript.

Field-specific reporting

Please select the one below that is the best fit for your research. If you are not sure, read the appropriate sections before making your selection.

☒ Life sciences ☐ Behavioural & social sciences ☐ Ecological, evolutionary & environmental sciences

For a reference copy of the document with all sections, see nature.com/documents/nr-reporting-summary-flat.pdf

Life sciences study design

All studies must disclose on these points even when the disclosure is negative.

Sample size	Samples were accumulated until expected significance was reached. Estimated sample sizes were retrospectively determined to achieve 80% power to detect expected effect sizes using Matlab.
Data exclusions	Samples or animals were excluded in the analysis in the following situations: (1) In figures 1d, 2b, and 4 where NOT-DTN projecting L5 neurons were targeted, mice were excluded if the injection of Retro-cre AAV virus, identified post hoc, went beyond the boundary of NOT-DTN and spread to surrounding areas; (2) In the analysis of OKR gain, trials in which video-oculography failed due to eye blinking or tears were excluded from analysis; (3) In figure 3f and i, 3 mice and 1 mouse, respectively, were excluded because the thinned skulls over the visual cortex became opaque, attenuating the effective power of the blue LED light reaching deep cortical layers; (4) In figure 6c, f, i, mice were excluded if the injections of retrograde Cre virus to the IO and/or the injection of Cre-dependent hM4Di/tdTomato or ArchT virus to the NOT-DTN failed, identified post hoc. Those criteria were pre-established.
Replication	Methods used in this study are widely available and reproducible. Experiments were replicated in multiple mice. The numbers of replications in individual experiments were indicated in the figures or legends. Our data showed that the findings were consistent across different mice. This study did not attempt to replicate previous findings.
Randomization	We randomly allocated mice for treatment groups and control groups.
Blinding	Experimenters who performed the experiments were not blinded to experimental conditions because all experiments required the knowledge of experimental designs. No blinding was used during data analysis but all of data were analyzed in the same way strictly.

Reporting for specific materials, systems and methods

We require information from authors about some types of materials, experimental systems and methods used in many studies. Here, indicate whether each material, system or method listed is relevant to your study. If you are not sure if a list item applies to your research, read the appropriate section before selecting a response.

Materials & experimental systems

n/a	Involved in the study
☐	☒ Antibodies
☒	☐ Eukaryotic cell lines
☒	☐ Palaeontology and archaeology
☐	☒ Animals and other organisms
☒	☐ Clinical data
☒	☐ Dual use research of concern
☒	☐ Plants

Methods

n/a	Involved in the study
☒	☐ ChIP-seq
☒	☐ Flow cytometry
☒	☐ MRI-based neuroimaging

Antibodies

Antibodies used

To staining cFos, rabbit anti-c-Fos (Supplier: Synaptic Systems, CAT: 226003, LOT:6-72) was used as the primary antibody, and Alexa Fluor 633 anti-rabbit (Supplier: Invitrogen, CAT: A-21070, LOT:2079350) was used as the secondary antibody. To reveal the the ON and OFF sublaminae of the IPL, goat anti-VAChT (Supplier: Millipore, CAT: ABN100, LOT: 3974859), goat anti-ChAT (Supplier: Millipore, CAT: AB144P, LOT: 3926290) were used as the primary antibodies, and Donkey anti-goat-Alexa Fluor 488 (Supplier: Invitrogen, CAT: A32814, LOT: XH354849) was used as the secondary antibody. To reveal the dendritic morphology of retinal ganglion cells labelled by RV-mCherry, rabbit anti-RFP (Supplier: abcam, CAT: ab62341, LOT: GR3281448-1) was used as the primary antibody and Donkey anti-rabbit-Alexa Fluor 594 (Supplier: Invitrogen, CAT: A32754, LOT: X1344353) was used as the secondary antibody.

Validation

The above commercial antibodies have been previously validated by the manufactures (as outlined on their websites). They were also well characterized in the literature.

rabbit anti-c-Fos (Supplier: Synaptic Systems, CAT: 226003)
https://sysy.com/product-factsheet/SySy_226003

AAlexa Fluor 633 anti-rabbit (Supplier: Invitrogen, CAT: A-21070)
https://www.thermofisher.com/antibody/product/Goat-anti-Rabbit-IgG-H-L-Cross-Adsorbed-Secondary-Antibody-Polyclonal/A-21070

goat anti-VAChT (Supplier: Millipore, CAT: ABN100)
https://www.emdmillipore.com/CA/en/product/Anti-Vesicular-Acetylcholine-Transporter-VAChT-Antibody,MM_NF-ABN100

goat anti-ChAT (Supplier: Millipore, CAT: AB144P)
https://www.emdmillipore.com/CA/en/product/Anti-Choline-Acetyltransferase-Antibody,MM_NF-AB144P

Donkey anti-goat-Alexa Fluor 488 (Supplier: Invitrogen, CAT: A32814)
https://www.thermofisher.com/antibody/product/Donkey-anti-Goat-IgG-H-L-Highly-Cross-Adsorbed-Secondary-Antibody-Polyclonal/A32814

rabbit anti-RFP (Supplier: abcam, CAT: ab62341)
https://www.abcam.com/products/primary-antibodies/rfp-antibody-ab62341.html

Donkey anti-rabbit-Alexa Fluor 594 (Supplier: Invitrogen, CAT: A32754)
https://www.thermofisher.com/antibody/product/Donkey-anti-Rabbit-IgG-H-L-Highly-Cross-Adsorbed-Secondary-Antibody-Polyclonal/A32754

Animals and other research organisms

Policy information about studies involving animals; ARRIVE guidelines recommended for reporting animal research, and Sex and Gender in Research

Laboratory animals

We used the following mouse lines: Ai14 tdTomato reporter111 (Jackson Laboratory #007914), VGAT-ChR2-EYFP112 (Jackson Laboratory #014548), C57BL/6J (Jackson Laboratory #000664), and CD-1 (Charles River #022). Since there was no report of sex dimorphism in the cortical modulation of OKR, the sex was not considered in the experimental design. Mice of both sexes were used for experiments. Experimental mice were bred by crossing homozygous VGAT-ChR2-EYFP, homozygous Ai14 or C57BL/6J males with wild-type CD-1 females. Mice were housed in a vivarium with a reversed light cycle (12h day/12h night), ambient temperature at 22° C, and humidity at 50%. CD-1 x C57BL/6J hybrid mice were used in experiments where wild-type mice were needed unless otherwise noted. The age of mice used in experiments ranged between 2-5 months.

Wild animals

The study did not involve wild animals.

Reporting on sex	Both male and female were used in all experiments.
Field-collected samples	No field collected samples were used in the study.
Ethics oversight	All experimental procedures performed in this study were approved by the Biological Sciences Local Animal Care Committee, in accordance with guidelines established by the University of Toronto Animal Care Committee and the Canadian Council on Animal Care (protocol # 20012152 and 20012125).

Note that full information on the approval of the study protocol must also be provided in the manuscript.
